# The genome sequence of the lesser earwig, *Labia minor *(Linnaeus, 1758)

**DOI:** 10.12688/wellcomeopenres.23739.1

**Published:** 2025-02-21

**Authors:** Liam M. Crowley, Mark G. Telfer, University of Oxford and Wytham Woods Genome Acquisition Lab, Darwin Tree of Life Barcoding collective, Wellcome Sanger Institute Tree of Life Management, Samples and Laboratory team, Wellcome Sanger Institute Scientific Operations: Sequencing Operations, Wellcome Sanger Institute Tree of Life Core Informatics team, Tree of Life Core Informatics collective, Darwin Tree of Life Consortium

**Affiliations:** 1 University of Oxford, Oxford, England, UK; 2 Independent researcher, Ventnor, Isle of Wight, England, UK

**Keywords:** Labia minor, lesser earwig, genome sequence, chromosomal, Dermaptera

## Abstract

We present a genome assembly from an individual male *Labia minor* (the lesser earwig; Arthropoda; Insecta; Dermaptera; Spongiphoridae). The genome sequence spans 604.50 megabases. Most of the assembly is scaffolded into 7 chromosomal pseudomolecules, including the X sex chromosome. Two mitochondrial scaffolds were also assembled.

## Species taxonomy

Eukaryota; Opisthokonta; Metazoa; Eumetazoa; Bilateria; Protostomia; Ecdysozoa; Panarthropoda; Arthropoda; Mandibulata; Pancrustacea; Hexapoda; Insecta; Dicondylia; Pterygota; Neoptera; Polyneoptera; Dermaptera; Neodermaptera; Epidermaptera; Forficuloidea; Spongiphoridae; *Labia*; *Labia minor* (Linnaeus, 1758) (NCBI:txid146840).

## Background

The Lesser Earwig ( *Labia minor*), a small earwig species measuring 5–7 mm in length, is characterised by its dull yellow-brown colour, visible hind wings, and slender pincers, which are slightly curved in males but lack teeth in both sexes. Its common name originates from Old English, combining "ēare" (ear) and "wicga" (insect) ( Watts, 2022).

Among the nearly 2,000 species of earwig found worldwide, only four are native to the United Kingdom: the Common, Lesser, Lesne’s, and Short-winged Earwigs ( Watts, 2022). *L. minor* has a more restricted range than the Common Earwig ( *Forficula auricularia*), but it occurs in approximately half of the UK and Ireland, excluding the most northerly regions ( NBN Atlas Partnership, 2024).

Originally described as *Forficula minor* by Carl Linnaeus in 1758, the species was reassigned to the genus *Labia* by William Elford Leach in 1815, making *L. minor* the type species of this genus.

The Lesser Earwig thrives in environments rich in decaying organic material, such as compost heaps, where the heat generated by decomposition provides favourable conditions. Like other earwigs, it displays remarkable maternal care, guarding its eggs and feeding the young for their first week or two after hatching—a rare behaviour among insects ( Meunier, 2024).

This genome represents the first sequenced species of Dermaptera by the Darwin Tree of Life project, offering new insights into the evolutionary and ecological diversity of this unique insect order.

## Genome sequence report

**Figure 1.**
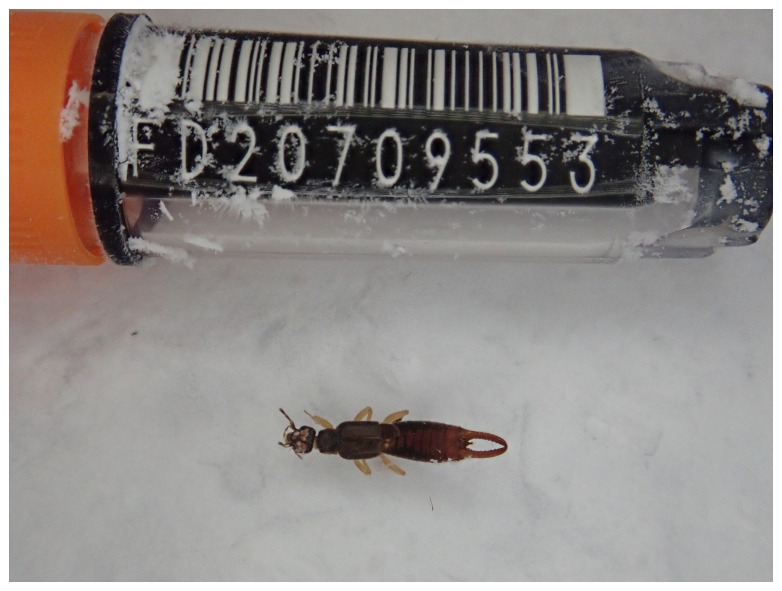
Photograph of the *Labia minor* (igLabMino1) specimen used for genome sequencing.

The genome of an adult male *Labia minor* ( Figure 1) was sequenced using Pacific Biosciences single-molecule HiFi long reads, generating a total of 14.74 Gb (gigabases) from 1.46 million reads, providing approximately 24-fold coverage. Chromosome conformation Hi-C sequencing produced 137.56 Gb from 910.97 million reads. Specimen and sequencing details are summarised in Table 1.

**Table 1.** Specimen and sequencing data for *Labia minor*.

Project information			
**Study title**	Labia minor (lesser earwig)		
**Umbrella BioProject**	PRJEB63625		
**Species**	*Labia minor*		
**BioSample**	SAMEA10107090		
**NCBI taxonomy ID**	146840		
Specimen information			
**Technology**	**ToLID**	**BioSample accession**	**Organism part**
**PacBio long read sequencing**	igLabMino1	SAMEA10200815	Whole organism
**Hi-C sequencing**	igLabMino1	SAMEA10200815	Whole organism
Sequencing information			
**Platform**	**Run accession**	**Read count**	**Base count (Gb)**
**Hi-C Illumina NovaSeq 6000**	ERR11641148	9.11e+08	137.56
**PacBio Sequel IIe**	ERR11641075	1.46e+06	14.74

**Figure 2.**
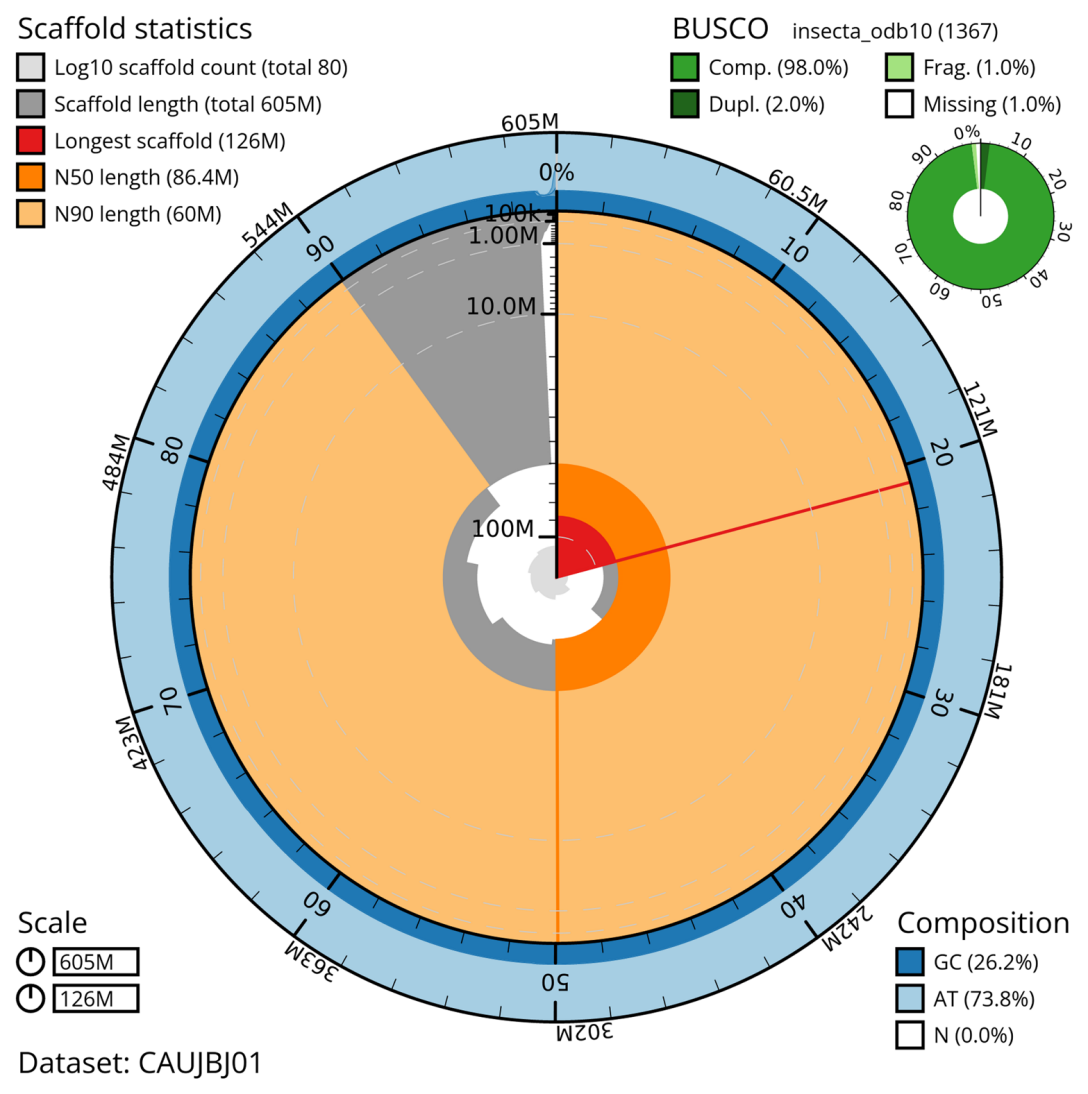
Genome assembly of *Labia minor*, igLabMino1.1: metrics. The BlobToolKit snail plot provides an overview of assembly metrics and BUSCO gene completeness. The circumference represents the length of the whole genome sequence, and the main plot is divided into 1,000 bins around the circumference. The outermost blue tracks display the distribution of GC, AT, and N percentages across the bins. Scaffolds are arranged clockwise from longest to shortest and are depicted in dark grey. The longest scaffold is indicated by the red arc, and the deeper orange and pale orange arcs represent the N50 and N90 lengths. A light grey spiral at the centre shows the cumulative scaffold count on a logarithmic scale. A summary of complete, fragmented, duplicated, and missing BUSCO genes in the insecta_odb10 set is presented at the top right. An interactive version of this figure is available at https://blobtoolkit.genomehubs.org/view/CAUJBJ01/dataset/CAUJBJ01/snail.

**Figure 3.**
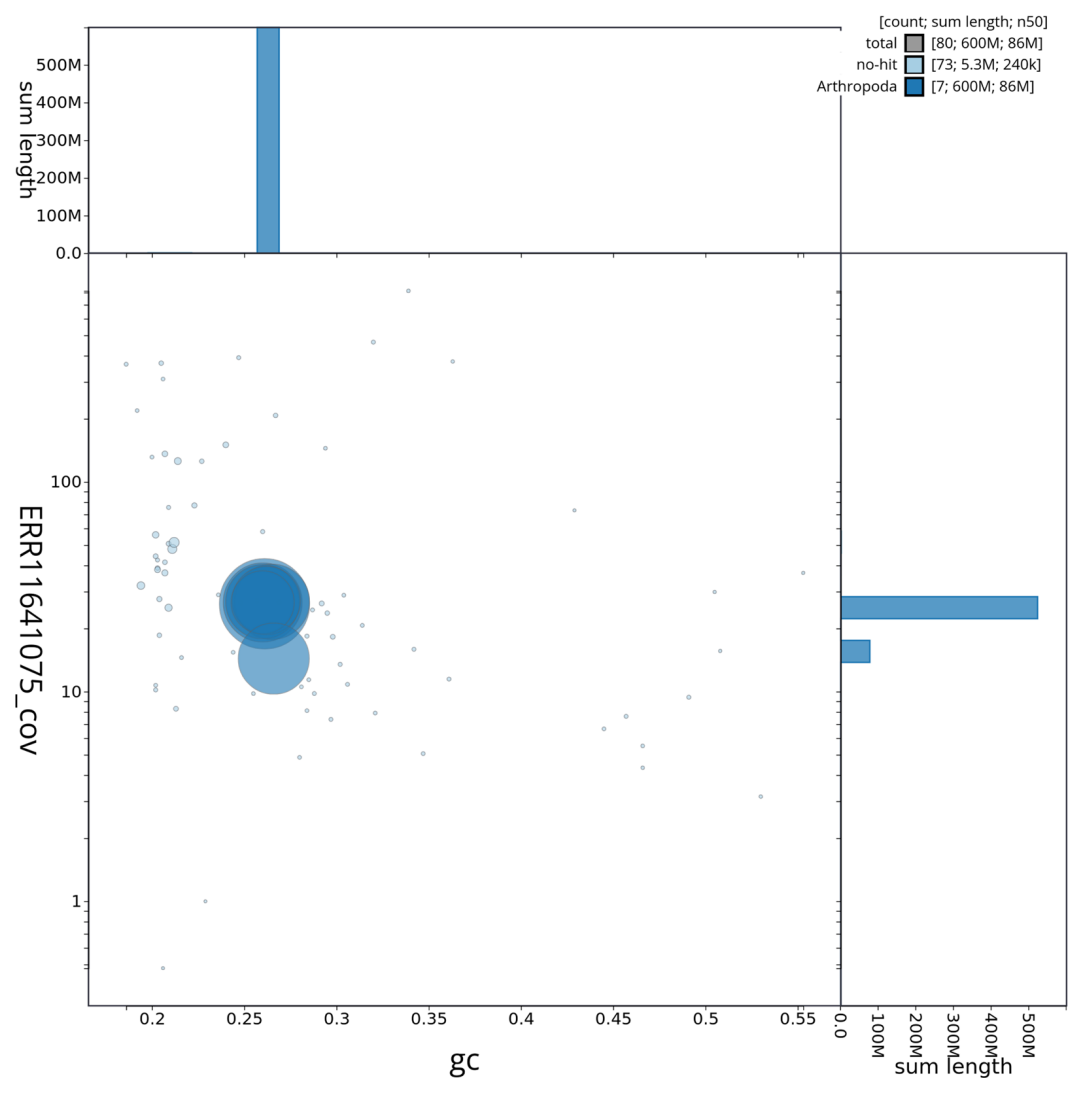
Genome assembly of *Labia minor*, igLabMino1.1: BlobToolKit GC-coverage plot. Sequences are coloured by phylum. Circles are sized in proportion to sequence length. Histograms show the distribution of sequence length sum along each axis. An interactive version of this figure is available at https://blobtoolkit.genomehubs.org/view/CAUJBJ01/dataset/CAUJBJ01/blob.

**Figure 4.**
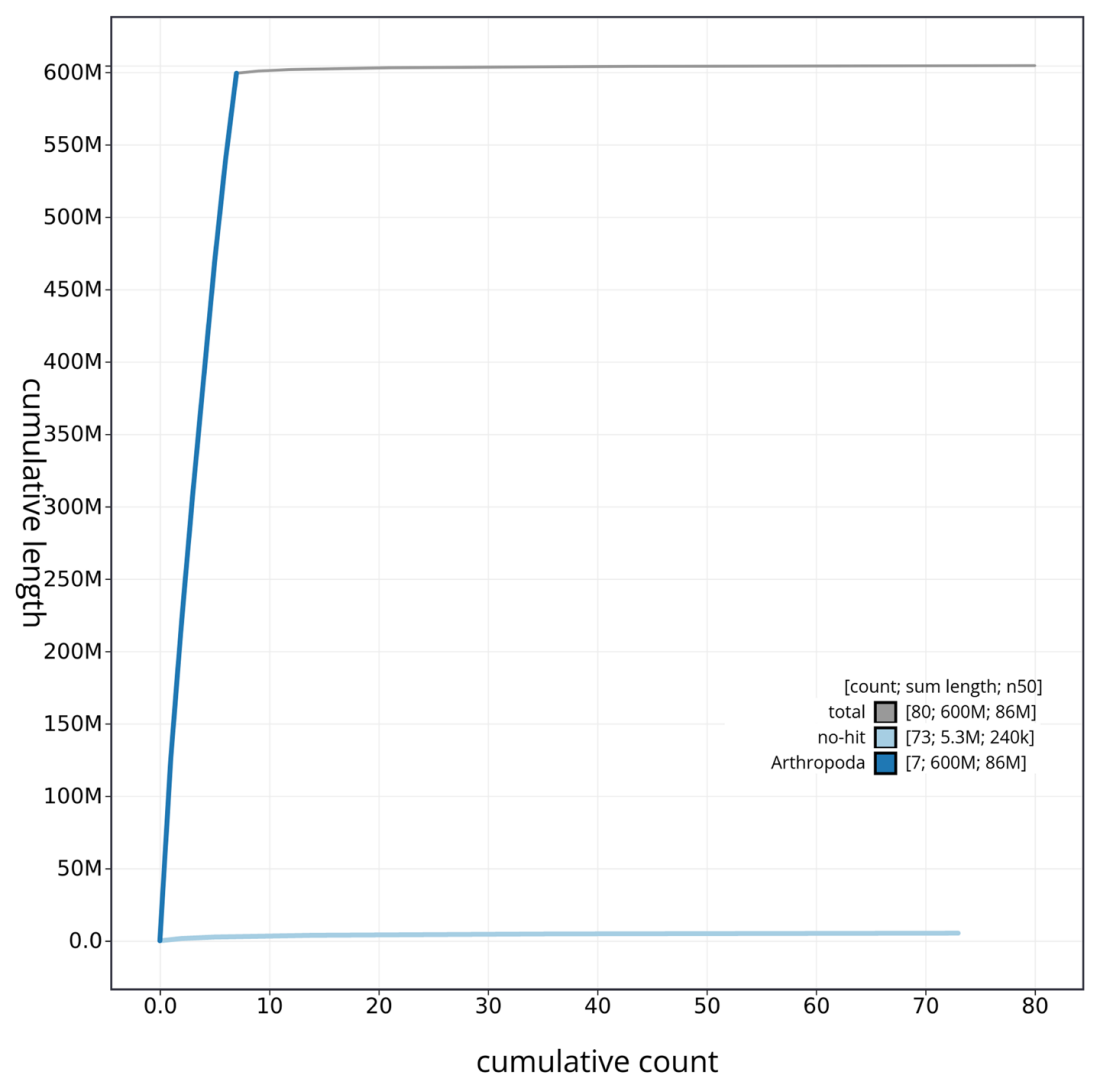
Genome assembly of *Labia minor* igLabMino1.1: BlobToolKit cumulative sequence plot. The grey line shows cumulative length for all sequences. Coloured lines show cumulative lengths of sequences assigned to each phylum using the buscogenes taxrule. An interactive version of this figure is available at https://blobtoolkit.genomehubs.org/view/CAUJBJ01/dataset/CAUJBJ01/cumulative.

**Figure 5.**
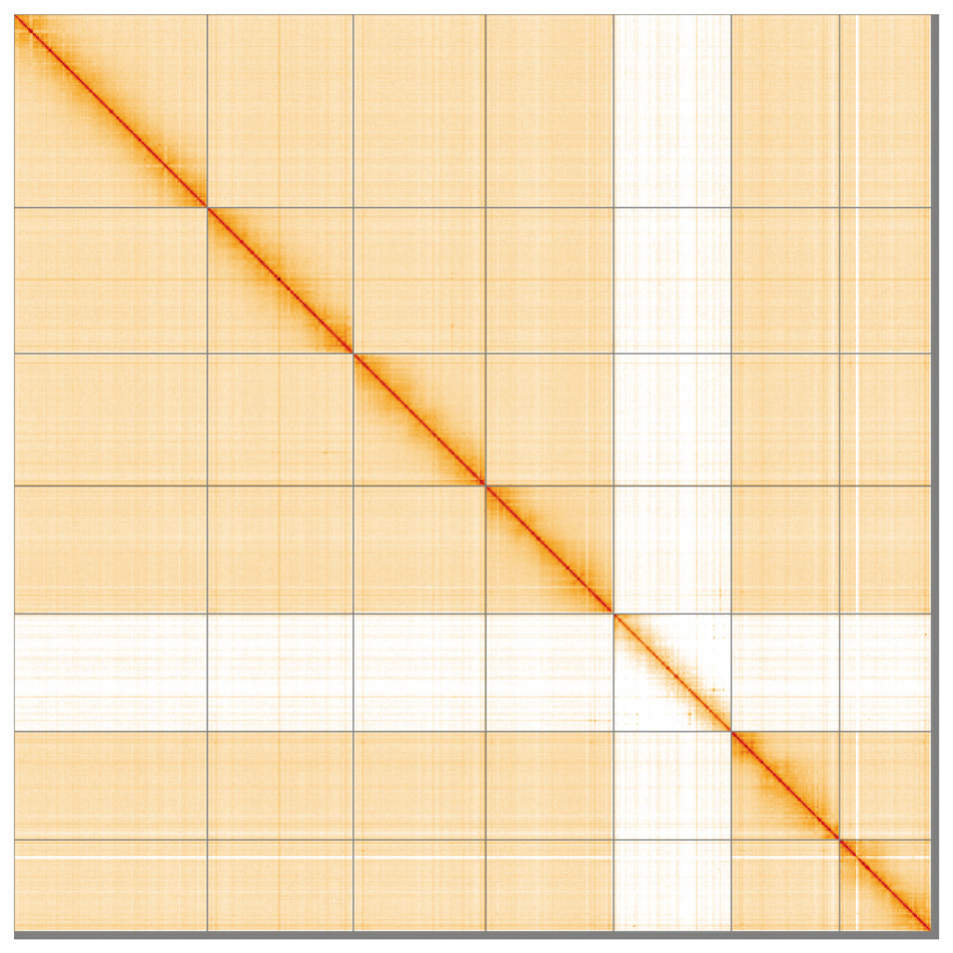
Genome assembly of *Labia minor* igLabMino1.1: Hi-C contact map of the igLabMino1.1 assembly, visualised using HiGlass. Chromosomes are shown in order of size from left to right and top to bottom. An interactive version of this figure may be viewed at

Manual assembly curation corrected 98 missing joins or mis-joins and three haplotypic duplications, reducing the scaffold number by 25.23%, and increasing the scaffold N50 by 1.81%. The final assembly has a total length of 604.50 Mb in 78 sequence scaffolds with a scaffold N50 of 86.4 Mb ( Table 2). The total count of gaps in the scaffolds is 404. The snail plot in Figure 2 provides a summary of the assembly statistics, while the distribution of assembly scaffolds on GC proportion and coverage is shown in Figure 3. The cumulative assembly plot in Figure 4 shows curves for subsets of scaffolds assigned to different phyla. Most (99.22%) of the assembly sequence was assigned to 7 chromosomal-level scaffolds, representing 6 autosomes and the X sex chromosome. Chromosome-scale scaffolds confirmed by the Hi-C data are named in order of size ( Figure 5; Table 3). Duration curation, the X chromosome was identified based on half coverage relative to autosomes. We expect to see a Y chromosome ( Morgan, 1928), but it does not appear to be present in the assembly.

**Table 2.** Genome assembly data for *Labia minor*, igLabMino1.1.

Genome assembly		
Assembly name	igLabMino1.1	
Assembly accession	GCA_963082975.1	
*Accession of alternate haplotype*	*GCA_963082615.1*	
Span (Mb)	604.50	
Number of contigs	484	
Number of scaffolds	78	
Longest scaffold (Mb)	126.26	
Assembly metrics *		*Benchmark*
Contig N50 length (Mb)	2.4	*≥ 1 Mb*
Scaffold N50 length (Mb)	86.4	*= chromosome N50*
Consensus quality (QV)	57.7	*≥ 40*
*k*-mer completeness	Primary: 92.25%; alternate: 77.83%; combined: 97.59%	*≥ 95%*
BUSCO **	C:98.0%[S:96.0%,D:2.0%], F:1.0%,M:1.0%,n:1,367	*S > 90%*, *D < 5%*
Percentage of assembly mapped to chromosomes	99.22%	*≥ 90%*
Sex chromosomes	X	*localised homologous pairs*
Organelles	Mitochondrial genome: None kb	*complete single alleles*

* Assembly metric benchmarks are adapted from column VGP-2020 of “Table 1: Proposed standards and metrics for defining genome assembly quality” from Rhie *et al.* (2021). ** BUSCO scores based on the insecta_odb10 BUSCO set using version 5.3.2. C = complete [S = single copy, D = duplicated], F = fragmented, M = missing, n = number of orthologues in comparison. A full set of BUSCO scores is available at https://blobtoolkit.genomehubs.org/view/CAUJBJ01/dataset/CAUJBJ01/busco.

**Table 3.** Chromosomal pseudomolecules in the genome assembly of *Labia minor*, igLabMino1.

INSDC accession	Name	Length (Mb)	GC%
OY720344.1	1	126.26	26.0
OY720345.1	2	95.4	26.0
OY720346.1	3	86.45	26.5
OY720347.1	4	83.48	26.0
OY720349.1	5	70.76	26.0
OY720350.1	6	60.0	26.0
OY720348.1	X	76.89	26.5
OY720351.1	MT1	0.01	36.0
OY720352.1	MT2	0.01	34.5

While not fully phased, the assembly deposited is of one haplotype. Contigs corresponding to an alternate haplotype have also been deposited. Two mitochondrial scaffolds were also assembled and can be found as a contig within the multifasta file of the genome submission.

The estimated Quality Value (QV) of the final assembly is 57.7. The *k*-mer completeness values were estimated as 92.25% for the primary assembly, 77.83% for the alternate haplotype and 97.59% for the combined primary and alternate haplotypes. The primary assembly has a BUSCO v5.3.2 completeness of 98.0% (single = 96.0%, duplicated = 2.0%), using the insecta_odb10 reference set ( *n* = 1,367).

## Methods

### Sample acquisition

An adult male *Labia minor* (specimen ID Ox001171, ToLID igLabMino1) was collected from Wytham Farm, Oxfordshire, UK (latitude 51.79, longitude –1.32) on 2021-04-13 by potting. The specimen was collected by Liam Crowley (University of Oxford) and identified by Mark Telfer (independent researcher) and preserved on dry ice.

The initial identification was verified by an additional DNA barcoding process according to the framework developed by Twyford *et al*. (2024). A small sample was dissected from the specimens and stored in ethanol, while the remaining parts of the specimen were shipped on dry ice to the Wellcome Sanger Institute (WSI). The tissue was lysed, the COI marker region was amplified by PCR, and amplicons were sequenced and compared to the BOLD database, confirming the species identification ( Crowley *et al.*, 2023). Following whole genome sequence generation, the relevant DNA barcode region is also used alongside the initial barcoding data for sample tracking at the WSI ( Twyford *et al.*, 2024). The standard operating procedures for Darwin Tree of Life barcoding have been deposited on protocols.io ( Beasley *et al.*, 2023).

### Nucleic acid extraction

The workflow for high molecular weight (HMW) DNA extraction at the Wellcome Sanger Institute (WSI) Tree of Life Core Laboratory includes a sequence of procedures: sample preparation; sample homogenisation, DNA extraction, fragmentation, and clean-up. In sample preparation, the igLabMino1 sample was weighed and dissected on dry ice ( Jay *et al.*, 2023).

Tissue from the whole organism was homogenised using a PowerMasher II tissue disruptor ( Denton *et al.*, 2023). HMW DNA was extracted using the Automated MagAttract v1 protocol ( Sheerin *et al.*, 2023). DNA was sheared into an average fragment size of 12–20 kb in a Megaruptor 3 system ( Todorovic *et al.*, 2023). Sheared DNA was purified by solid-phase reversible immobilisation ( Strickland *et al.*, 2023), using AMPure PB beads to eliminate shorter fragments and concentrate the DNA. The concentration of the sheared and purified DNA was assessed using a Nanodrop spectrophotometer and Qubit Fluorometer using the Qubit dsDNA High Sensitivity Assay kit. Fragment size distribution was evaluated by running the sample on the FemtoPulse system.

### Hi-C sample preparation

Tissue from the sample was processed using the Arima-HiC v2 kit at the WSI Scientific Operations core. In brief, 20–50 mg of frozen tissue (stored at –80 °C) was fixed, and the DNA crosslinked using a TC buffer with 22% formaldehyde concentration. After crosslinking the tissue was homogenised using the Diagnocine Power Masher-II and BioMasher-II tubes and pestles. Following the Arima-HiC v2 kit manufacturer's instructions, crosslinked DNA was digested using a restriction enzyme master mix. The 5’-overhangs were filled in and labelled with biotinylated nucleotides and proximally ligated. An overnight incubation was carried out for enzymes to digest remaining proteins and for crosslinks to reverse. A clean up was performed with SPRIselect beads prior to library preparation. Additionally, the biotinylation percentage was estimated using the Qubit Fluorometer v4.0 (Thermo Fisher Scientific) and Qubit HS Assay Kit and Arima-HiC v2 QC beads.

### Library preparation and sequencing

***PacBio HiFi***

Libraries were prepared using the PacBio Express Template Preparation Kit v2.0 (Pacific Biosciences, California, USA) as per the manufacturer's instructions. The kit includes the reagents required for removal of single-strand overhangs, DNA damage repair, end repair/A-tailing, adapter ligation, and nuclease treatment. Library preparation also included a library purification step using AMPure PB beads (Pacific Biosciences, California, USA) and size selection step to remove templates <3kb using AMPure PB modified SPRI. DNA concentration was quantified using the Qubit Fluorometer v2.0 (Thermo Fisher Scientific) and Qubit HS Assay Kit and the final library fragment size analysis was carried out using the Agilent Femto Pulse Automated Pulsed Field CE Instrument (Agilent Technologies).

Samples were sequenced using the Sequel IIe system (Pacific Biosciences, California, USA). The concentration of the library loaded onto the Sequel IIe was in the range 40–135 pM. The SMRT link software, a PacBio web-based end-to-end workflow manager, was used to set-up and monitor the run, as well as perform primary and secondary analysis of the data upon completion.

***Hi-C***

For Hi-C library preparation, DNA was fragmented using the Covaris E220 sonicator (Covaris) and size selected using SPRISelect beads to 400 to 600 bp. The DNA was then enriched using the Arima-HiC v2 kit Enrichment beads. Using the NEBNext Ultra II DNA Library Prep Kit (New England Biolabs) for end repair, a-tailing, and adapter ligation. This uses a custom protocol which resembles the standard NEBNext Ultra II DNA Library Prep protocol but where library preparation occurs while DNA is bound to the Enrichment beads. For library amplification, 10–16 PCR cycles were required, determined by the sample biotinylation percentage. The Hi-C sequencing was performed using paired-end sequencing with a read length of 150 bp on an Illumina NovaSeq 6000.

### Genome assembly, curation and evaluation

***Assembly***

The original assembly of HiFi reads was performed using Hifiasm ( Cheng *et al.*, 2021) with the --primary option. Haplotypic duplications were identified and removed with purge_dups ( Guan *et al.*, 2020). Hi-C reads are further mapped with bwa-mem2 ( Vasimuddin *et al.*, 2019) to the primary contigs, which are further scaffolded using the provided Hi-C data ( Rao *et al.*, 2014) in YaHS ( Zhou *et al.*, 2023) using the --break option. Scaffolded assemblies are evaluated using Gfastats ( Formenti *et al.*, 2022), BUSCO ( Manni *et al.*, 2021) and MERQURY.FK ( Rhie *et al.*, 2020).

The mitochondrial genome was assembled using MBG ( Rautiainen & Marschall, 2021) from PacBio HiFi reads mapping to related genomes. A representative sequence was selected for each from the graph based on read coverage, contig size, and its alignments to the related genomes, then MitoHiFi ( Uliano-Silva *et al.*, 2023) was run on this sequence for circularisation and annotation with MitoFinder ( Allio *et al.*, 2020).

***Assembly curation***

The assembly was decontaminated using the Assembly Screen for Cobionts and Contaminants (ASCC) pipeline (article in preparation). Flat files and maps used in curation were generated in TreeVal ( Pointon *et al.*, 2023). Manual curation was primarily conducted using PretextView ( Harry, 2022), with additional insights provided by JBrowse2 ( Diesh *et al.*, 2023) and HiGlass ( Kerpedjiev *et al.*, 2018). Scaffolds were visually inspected and corrected as described by Howe *et al*. (2021). Any identified contamination, missed joins, and mis-joins were corrected, and duplicate sequences were tagged and removed. The curation process is documented at https://gitlab.com/wtsi-grit/rapid-curation (article in preparation).

***Assembly quality assessment***

The Merqury.FK tool ( Rhie *et al.*, 2020), run in a Singularity container ( Kurtzer *et al.*, 2017), was used to evaluate *k*-mer completeness and assembly quality for the primary and alternate haplotypes using the *k*-mer databases ( *k* = 31) that were computed prior to genome assembly. The analysis outputs included assembly QV scores and completeness statistics.

A Hi-C contact map was produced for the final version of the assembly. The Hi-C reads were aligned using bwa-mem2 ( Vasimuddin *et al.*, 2019) and the alignment files were combined using SAMtools ( Danecek *et al.*, 2021). The Hi-C alignments were converted into a contact map using BEDTools ( Quinlan & Hall, 2010) and the Cooler tool suite ( Abdennur & Mirny, 2020). The contact map was visualised in HiGlass ( Kerpedjiev *et al.*, 2018).

The genome was also analysed within the BlobToolKit environment ( Challis *et al.*, 2020) and BUSCO scores ( Manni *et al.*, 2021) were calculated.

Table 4 contains a list of relevant software tool versions and sources.

**Table 4.** Software tools: versions and sources.

Software tool	Version	Source
BlobToolKit	4.2.1	https://github.com/blobtoolkit/blobtoolkit
BUSCO	5.3.2	https://gitlab.com/ezlab/busco
Hifiasm	0.16.1-r375	https://github.com/chhylp123/hifiasm
HiGlass	1.11.6	https://github.com/higlass/higlass
Merqury	MerquryFK	https://github.com/thegenemyers/MERQURY.FK
MBG	3a3c79c24169c8155492057ff7bfa7acc4e3fcd8	https://github.com/maickrau/MBG
MitoHiFi	2	https://github.com/marcelauliano/MitoHiFi
PretextView	0.2	https://github.com/wtsi-hpag/PretextView
purge_dups	1.2.3	https://github.com/dfguan/purge_dups
YaHS	1.1a.2	https://github.com/c-zhou/yahs

### Wellcome Sanger Institute – Legal and Governance

The materials that have contributed to this genome note have been supplied by a Darwin Tree of Life Partner. The submission of materials by a Darwin Tree of Life Partner is subject to the **‘Darwin Tree of Life Project Sampling Code of Practice’**, which can be found in full on the Darwin Tree of Life website here. By agreeing with and signing up to the Sampling Code of Practice, the Darwin Tree of Life Partner agrees they will meet the legal and ethical requirements and standards set out within this document in respect of all samples acquired for, and supplied to, the Darwin Tree of Life Project.

Further, the Wellcome Sanger Institute employs a process whereby due diligence is carried out proportionate to the nature of the materials themselves, and the circumstances under which they have been/are to be collected and provided for use. The purpose of this is to address and mitigate any potential legal and/or ethical implications of receipt and use of the materials as part of the research project, and to ensure that in doing so we align with best practice wherever possible. The overarching areas of consideration are:

•   Ethical review of provenance and sourcing of the material

•   Legality of collection, transfer and use (national and international)

Each transfer of samples is further undertaken according to a Research Collaboration Agreement or Material Transfer Agreement entered into by the Darwin Tree of Life Partner, Genome Research Limited (operating as the Wellcome Sanger Institute), and in some circumstances other Darwin Tree of Life collaborators.

## Data Availability

European Nucleotide Archive: Labia minor (lesser earwig). Accession number PRJEB63625; https://identifiers.org/ena.embl/PRJEB63625. The genome sequence is released openly for reuse. The *Labia minor* genome sequencing initiative is part of the Darwin Tree of Life (DToL) project. All raw sequence data and the assembly have been deposited in INSDC databases. The genome will be annotated using available RNA-Seq data and presented through the Ensembl pipeline at the European Bioinformatics Institute. Raw data and assembly accession identifiers are reported in Table 1 and Table 2.

## Author information

Members of the University of Oxford and Wytham Woods Genome Acquisition Lab are listed here: https://doi.org/10.5281/zenodo.12157525.

Members of the Darwin Tree of Life Barcoding collective are listed here: https://doi.org/10.5281/zenodo.12158331

Members of the Wellcome Sanger Institute Tree of Life Management, Samples and Laboratory team are listed here: https://doi.org/10.5281/zenodo.12162482.

Members of Wellcome Sanger Institute Scientific Operations: Sequencing Operations are listed here: https://doi.org/10.5281/zenodo.12165051.

Members of the Wellcome Sanger Institute Tree of Life Core Informatics team are listed here: https://doi.org/10.5281/zenodo.12160324.

Members of the Tree of Life Core Informatics collective are listed here: https://doi.org/10.5281/zenodo.12205391.

Members of the Darwin Tree of Life Consortium are listed here: https://doi.org/10.5281/zenodo.4783558.
