# Supplementary material for: The genome sequence of the lesser earwig, Labia minor (Linnaeus, 1758)
Source: Wellcome Open Res. 2025 Feb 21;10:90. [Version 1] doi: 10.12688/wellcomeopenres.23739.1 (PMC11937785; doi:10.12688/wellcomeopenres.23739.1)
Supplement: Peer review history

# Reviewer report: Reviewer response for version 1

**DOI:** 10.21956/wellcomeopenres.26187.r119289

**Published:** 2025-03-25

**Authors:** Alice Dennis

**Affiliations:** 1 University of Namur, Namur, Belgium

This is a nice report of the genome assembly of *L. minor*. The methods and results are clear. Sequencing was done by HiFi reads, followed by HiC scaffolding. Manual curation has led to a chromosome-level assembly, which will be useful for future research.

**I just have a few minor questions:**

 1. Why do you think the Y-chromosome was not located? Is this expected to be very small, for example?

 2. The blobplot has a scattering of "no hit" scaffolds. Did you explore what these are? This appears to be after you have run a co-biont/contaminant screening, so I think it would be worthwhile to explore these further to see if they should be removed.

 3. Are there any related species that have been sequenced, to provide a comparison of the genome size, GC content, and other stats?

Are sufficient details of methods and materials provided to allow replication by others?

Yes

Is the rationale for creating the dataset(s) clearly described?

Yes

Are the datasets clearly presented in a useable and accessible format?

Yes

Are the protocols appropriate and is the work technically sound?

Yes

Reviewer Expertise:

Ecology, Evolution, Adaptation, and Genomics

I confirm that I have read this submission and believe that I have an appropriate level of expertise to confirm that it is of an acceptable scientific standard.

# Reviewer report: Reviewer response for version 1

**DOI:** 10.21956/wellcomeopenres.26187.r119283

**Published:** 2025-02-27

**Authors:** İnci Şahin Negiş

**Affiliations:** 1 Selcuk University, Selçuklu-Konya, Turkey

This manuscript reports the genome sequencing of an adult male *Labia minor* using Pacific Biosciences HiFi long-read technology. A total of 14.74 Gb of sequence data was generated, providing approximately 24× coverage, while Hi-C sequencing yielded 137.56 Gb of data. Following manual curation, errors such as mis-joins and haplotypic duplications were corrected, reducing the number of scaffolds by 25.23% and improving the scaffold N50 to 86.4 Mb. The final assembly spans 604.50 Mb across 78 scaffolds, with 99.22% of the sequence assigned to seven chromosome-level scaffolds, including six autosomes and the X chromosome. Although the presence of a Y chromosome has been previously suggested, it was not detected in this assembly.

 The study presents high-quality genomic data in a clear and structured manner. However, incorporating morphological diagnostic characteristics in the materials and methods section could enhance accessibility for readers less familiar with *Labia minor*, providing a broader taxonomic context.

Are sufficient details of methods and materials provided to allow replication by others?

Yes

Is the rationale for creating the dataset(s) clearly described?

Yes

Are the datasets clearly presented in a useable and accessible format?

Yes

Are the protocols appropriate and is the work technically sound?

Yes

Reviewer Expertise:

molecular entomology

I confirm that I have read this submission and believe that I have an appropriate level of expertise to confirm that it is of an acceptable scientific standard.
